# Use of *Aureobasidium* in a sustainable economy

**DOI:** 10.1007/s00253-024-13025-5

**Published:** 2024-02-13

**Authors:** Stephanie Rensink, Elke J. van Nieuwenhuijzen, Michael F. Sailer, Christian Struck, Han A. B. Wösten

**Affiliations:** 1 Department of Biology, Microbiology, Utrecht University, Padualaan 8, 3584 CH Utrecht, the Netherlands; 2 Department of Business, Building and Technology, Sustainable Building Technology, Saxion University of Applied Sciences, M.H. Tromplaan 28, 7513 AB Enschede, the Netherlands; 3 Faculty of Technology, Amsterdam University of Applied Sciences, Rhijnspoorplein 2, 1091 GC Amsterdam, The Netherlands

**Keywords:** *Aureobasidium*, Fungus, Biofilm, Coating, Wood protection, Engineered living material

## Abstract

**Abstract:**

*Aureobasidium* is omnipresent and can be isolated from air, water bodies, soil, wood, and other plant materials, as well as inorganic materials such as rocks and marble. A total of 32 species of this fungal genus have been identified at the level of DNA, of which *Aureobasidium pullulans* is best known. *Aureobasidium* is of interest for a sustainable economy because it can be used to produce a wide variety of compounds, including enzymes, polysaccharides, and biosurfactants. Moreover, it can be used to promote plant growth and protect wood and crops. To this end, *Aureobasidium* cells adhere to wood or plants by producing extracellular polysaccharides, thereby forming a biofilm. This biofilm provides a sustainable alternative to petrol-based coatings and toxic chemicals. This and the fact that *Aureobasidium* biofilms have the potential of self-repair make them a potential engineered living material avant la lettre.

**Key points:**

•*Aureobasidium produces products of interest to the industry*

•*Aureobasidium can stimulate plant growth and protect crops*

•*Biofinish of A. pullulans is a sustainable alternative to petrol-based coatings*

•*Aureobasidium biofilms have the potential to function as engineered living materials*

## Introduction

The growing world population causes environmental problems such as resource depletion, biodiversity loss, and pollution (Geissdoerfer et al. 2017; Maja and Ayano 2021). For instance, it drives the expansion of agricultural activity (Toop et al. 2017) and the building industry. The latter consumes large volumes of natural resources (e.g., sand, gravel, and oil) and energy and produces high amounts of CO_2_ and solid waste (Benachio et al. 2020). Therefore, there is a huge urgency to shift to a sustainable economy to reduce human impact on the environment.

The building industry relies on coatings to prevent the breakdown of building materials. These coatings are often based on non-renewable oil and contain toxic chemicals such as chromated copper arsenate, creosote, pentachlorophenol, or heavy metal combinations (Morrell 2017). Similarly, food products are often treated with chemicals such as copper-based fungicides to prevent colonization with pathogens or spoilage organisms (Lamichhane et al. 2018). The fungus *Aureobasidium* can contribute to the transition to a sustainable economy by providing a sustainable coating for wood and food products. Also, *Aureobasidium* can be used as a cell factory for the production of enzymes and other natural compounds that can replace non-sustainable chemicals.

This review describes the diversity and ecological niches of *Aureobasidium*. Moreover, it describes its life cycle, its (potential) use to protect products against deterioration, and as a (potential) source for of biobased molecules such as enzymes, biosurfactants, melanin, siderophores, gluconic acid, and the polysaccharides pullulan and β-glucan (Brumano et al. 2017; Canete-Rodriguez et al. 2016; Wang et al. 2022b). Furthermore, we describe the perspective of exploiting and expanding *Aureobasidium* as a component of a sustainable engineered living material with its biofilm on wood as a first example.

## The genus *Aureobasidium*

*Aureobasidium* belongs to the phylum Ascomycota and the order Dothideales (Thambugala et al. 2014). Previously, *Aureobasidium* belonged to the *Dothideaceae* family (Schoch et al. 2006) but was reclassified in 2014 to belong to the *Aureobasidiaceae*. The latter family also includes the genera *Kabatiella*, *Pseudoseptoria*, *Saccothecium*, and the species *Selenophoma mahoniae* and *Columnosphaeria fagi* (Thambugala et al. 2014). *Aureobasidium* is described as mildew or blue or black stain (de Hoog 1993) and is popularly known as black yeast (Singh et al. 2015). Species of this genus can be found on all continents (Loque et al. 2010; Merín et al. 2011; Onetto et al. 2020; Peterson et al. 2013; van Nieuwenhuijzen et al. 2016; Woody et al. 2003) and have been isolated from air, water, and diverse (in)organic outdoor and indoor materials such as soil, phylloplanes, wood, rocks, marble, dishwashers, washing machines, house dust, and food (Babič et al. 2015; Humphries et al. 2017; Jiang et al. 2018; Li et al. 2015; van Nieuwenhuijzen 2014; Wang et al. 2022a; Zupancic et al. 2016). Currently, 32 DNA-identified *Aureobasidium* species are known (Table 1). These species include the best known *Aureobasidium species, Aureobasidium pullulans,* as well as the most recently identified species *Aureobasidium insectorum*, *Aureobasidium planticola*, *Aureobasidium motuoense*, and *Aureobasidium intercalariosporum* (Arnaud 1918; Arzanlou and Khodaei 2012; Ashish and Pratibha 2018; Barr 2001; Bills et al. 2012; Ciferri et al. 1957; Cooke 1962; Crous et al. 2021, 2011; de Hoog and Hermanides-Nijhof 1977a, 1977b; Gostinčar et al. 2014; Inamdar et al. 2019; Jia et al. 2019; Jiang et al. 2021, 2019; Lee et al. 2021; Nasr et al. 2018; Onetto et al. 2020; Peterson et al. 2013; Ramaley 1992; Wang et al. 2022a; Wu et al. 2023). An additional 15 species have been identified based on morphology (Table 2) (Cooke 1962; Crisan and Hodisan 1964; Della Torre 1963; de Hoog and Hermanides-Nijhof 1977a; Pande and Ghate 1985; Richardson and Pitkäranta 2011). Various loci (i.e., the internal transcribed spacer (ITS) rDNA, intergenic spacer 1, translation elongation factor-1α, β-tubulin, large ribosomal subunit (LSU), and RNA polymerase II) have been used for the phylogeny of *Aureobasidium* (Crous et al. 2011; Gostinčar et al. 2014; Manitchotpisit et al. 2009; Peterson et al. 2013; Wang et al. 2022a; Zalar et al. 2008). *Kabatiella* and *Aureobasidium* are closely related based on morphology and DNA sequences, which makes them difficult to distinguish (Bills et al. 2012; Crous et al. 2011). In fact, *Kabatiella lini* is now proposed to be part of the *Aureobasidium* clade (Thambugala et al. 2014). The same holds for *Selenophoma mahoniae* and *Columnosphaeria fagi*.

**Table 1:** DNA-identified *Aureobasidium* species

Species name	Synonym	Effective publication (according to the reference or MycoBank on 1 February 2023)		Source	Location	Mycobank, CBS, strain number	Reference
		Name	Year				
*Aureobasidium acericola*		D. Hyeon Lee, J.Y. Oh, and Jong Kyu Lee	2021	Leaves of *Acer pseudosieboldianum*	Korea	MB836925	Lee et al. (2021)
*Aureobasidium aerium*		N. Jiang	2022	Air	Beijing, China	MB843527, CFCC 50324	Wang et al. (2022a)
*Kabatiella bupleuri*	*Aureobasidium bupleuri*	(Bills) Haelewaters and Aime	2021	Dead *Bupleurum gibraltarium* flower rachises	Spain	MB835676, CBS 131304	Bills et al. (2012)
*Aureobasidium castaneae*		C.M. Tian and N. Jiang	2021	*Castanea henryi* leaves	China	MB838314JJ7-3	Jiang et al. (2021)
*Kabatiella caulivora*	*Aureobasidium caulivorum*	(Kirchn.) W.B. Cooke	1962	*Trifolium incarnatum*	USA	MB326817, CBS 242.64	Cooke (1962)
*Aureobasidium hainanensis*	*Aureobasidium pullulans strain P6*	S-L. Jia, Y. Ma, Z. Chi, G-L. Liu, Z. Hu, and Z-M. Chi	2019	*Kandelia candel* leaf	China	RZIQ01000000	Jia et al. (2019)
*Kabatiella harpospora*	*Aureobasidium harposporum*	(Bres. and Sacc.) Hermanides-Nijhof	1977	*Viscum album*	Madrid, Spain	MB309380, CBS 122914	de Hoog and Hermanides-Nijhof (1977a)
*Aureobasidium insectorum*		Q.M. Wang, F. Wu, and M.M. Wang	2023	Spittle insects	China	OP856707, OP857208	Wu et al. (2023)
*Aureobasidium intercalariosporum*		Q.M. Wang, F. Wu, and M.M. Wang	2023	Leaf	China	OP856703, OP857205	Wu et al. (2023)
*Aureobasidium iranianum*		Arzanlou and Khodaei	2012	Bamboo	Iran	MB800705, CCTU 268	Arzanlou and Khodaei (2012)
*Aureobasidium khasianum*		J. Pratibha and Prabhug	2018	Decaying leaves of *Wightia speciosissima*	India	MB828278AVP 109	Ashish and Pratibha (2018)
*Aureobasidium leucospermi*		Crous	2011	*Leucospermum conocarpodendron* leaves	Stellenbosch, South Africa	MB560556, CBS 130593	Crous et al. (2011)
*Kabatiella lini*	*Aureobasidium lini*	(Laff.) Hermanides-Nijhof	1977	*Linum usitatissimum*	UK	MB283371, CBS 125.21	de Hoog and Hermanides-Nijhof (1977a)
*Aureobasidium mangrovei*		S. Nasr	2018	Healthy *Avicennia marina* plant	Qeshm Island, Iran	MB823444, IBRC M 30265	Nasr et al. (2018)
*Aureobasidium melanogenum*		(Hermanides-Nijhof) Zalar, Gostincar, and Gunde-Cimerman	2014	N/A	N/A	MB807698, CBS 105.22	Gostinčar et al. (2014)
*Aureobasidium* confer (cf.)* melanogenum*	*Aureobasidium melanogenum*	(de Bary) G. Arnaud	1918	Public fountain	Bangkok, Thailand	CBS 110374	Arnaud (1918) and van Nieuwenhuijzen et al. (2016)
*Aureobasidium microtermitis*		S. Tiwari and A. Baghela	2021	*Microtermes* sp. Termite gut	Gujarat, Rajpipla district, India	MB839078, GTS2.7	Crous et al. (2021)
*Aureobasidium motuoense*		Q.M. Wang, F. Wu, and M.M. Wang	2023	Leaf	China	OP856710, OP857211	Wu et al. (2023)
*Aureobasidium mustum*		C. Onetto, S. Schmidt, M. Roach, and A. Borneman	2020	Fresh grape juice	South Australia	MB836845	Onetto et al. (2020)
*Aureobasidium namibiae*		(Zalar, de Hoog and Gunde-Cimerman) Zalar, Gostincar, and Gunde-Cimerman	2014	Dolomitic marble	Namib Desert, Namibia	MB807701, CBS 147.97	Gostinčar et al. (2014)
*Aureobasidium pini*		C.M. Tian and N. Jiang	2019	Pine needle	China	MB828664, CFCC 52778	Jiang et al. (2019)
*Aureobasidium planticola*		Q.M. Wang, F. Wu, and M.M. Wang	2023	Leaf	China	OP856711, OP857212	Wu et al. (2023)
*Aureobasidium proteae*		(Joanne E. Taylor and Crous) Joanne E. Taylor and Crous	2011	Leaves of *Protea* cv. ‘*Sylvia*’	South Africa	MB560557, CBS 114273	Crous et al. (2011)
*Aureobasidium pullulans*		(De Bary) G. Arnaud ex Cif., Ribaldi, and Corte	1957	Vitis vinifera, fruit	Beaujolais, Beaujeu, France	MB508998, CBS 584.75	Ciferri et al. (1957) and Gostinčar et al. (2014)
*Aureobasidium subglaciale*		(Zalar, de Hoog and Gunde-Cimerman) Zalar, Gostincar, and Gunde-Cimerman	2014	Subglacial ice from sea water	Kongsvegen, Svalbard, Norway	MB807700, CBS 123387	Gostinčar et al. (2014)
*Aureobasidium thailandense*		S. W. Peterson, Manitchotpisit, and Leathers	2013	Wood surface	Prachuapkhirikhan, Thailand	MB801148, NRRL 58543	Peterson et al. (2013)
*Aureobasidium tremulum*		Inamdar, Roh. Sharma, and Adhapure	2019	Culture contaminant in a laboratory	Aurangabad, Maharashtra, India	MB829941, ATS4.2	Inamdar et al. (2019)
*Aureobasidium uvarum*		C. Onetto, S. Schmidt, M. Roach, and A. Borneman	2020	Fresh grape juice	South Australia	MB836846	Onetto et al. (2020)
*Aureobasidium vineae*		C. Onetto, S. Schmidt, M. Roach, and A. Borneman	2020	Fresh grape juice	South Australia	MB836849	Onetto et al. (2020)
*Kabatiella zeae*	*Aureobasidium zeae*	(Narita and Y. Hirats.) Dingley	1973	Leaf of *Zea mays*	Kiel-Kitzeberg, Germany	MB283372, CBS 767.71	(de Hoog and Hermanides-Nijhof 1977b)
*Columnosphaeria fagi*		(H.J. Huds.) M.E. Barr	2001	Leaf of *Populus* sp.	UK	MB489000, CBS 171.93	Barr (2001)
*Selenophoma mahoniae*		A.W. Ramaley	1992	Leaf of *Mahonia repens*	USA	MB355521, CBS 388.92	Ramaley (1992)

*N/A*, not available

**Table 2:** Morphology-identified *Aureobasidium* species

Species name	Synonym	Name of effective publication	Year of effective publication	Source	Location	Mycobank MB No	Reference
*Aureobasidium aleuritis*	*Kabatiella aleuritis*	(Vassiljevsky) Hermanides-Nijhof	1977	Dying *Aleurites fordii* leaves	Russia	309377	Cooke (1962) and de Hoog and Hermanides-Nijhof (1977a)
*Aureobasidium apocryptum*	*Gloeosporium apocryptum*	(Ellis and Everh.) Hermanides-Nijhof	1977	Leaves of *Acer dasycarpum, A. negundo*, *A. saccharinum*, *A. saccharum, A. tataricum*, and *A. platanoides*	USA and Russia	309378	Cooke (1962) and de Hoog and Hermanides-Nijhof (1977a)
*Aureobasidium australiense*		McAlpine	1896	N/A	N/A	501787	Richardson and Pitkäranta (2011)
*Aureobasidium dalgeri*	*Kabatiella dalgeri*	(M. Morelet) Hermanides-Nijhof	1977	Dead *Eucalyptus* leaves	Saroula, Tunesia	309379	de Hoog and Hermanides-Nijhof (1977a)
*Aureobasidium indicum*		A. Pande and Ghate	1985	N/A	N/A	103074	Pande and Ghate (1985) and Richardson and Pitkäranta (2011)
*Aureobasidium lilii*		Crisan and Hodisan	1964	Plant	N/A	326819	Crisan and Hodisan (1964) and Richardson and Pitkäranta (2011)
*Aureobasidium microstromoides*	*Gloeosporium microstromoides*	(Moesz) W.B. Cooke	1962	*Catalpa bignonioides* capsules	Hungary	326822	Cooke (1962)
*Aureobasidium nigricans*	*Kabatiella nigricans*	(G.F. Atk. and Edgerton) W.B. Cooke	1962	*Vicia sativa*	N/A	326823	Cooke (1962)
*Aureobasidium nigrum*	*Torula dematia*	(Marpmann) Cif. and Dalla Torre	1963	N/A	N/A	326824	Della Torre (1963) and Richardson and Pitkäranta (2011)
*Aureobasidium prunicola*		(Ellis and Everh.) Hermanides-Nijhof	1977	*Prunus virginiana* leaves	Racine, Wisconsin, USA	309382	de Hoog and Hermanides-Nijhof (1977a)
*Aureobasidium ribis*	*Kabatiella ribis*	(Vassiljevsky) Hermanides-Nijhof	1977	*Ribes nigrum* leaves	N/A	309384	de Hoog and Hermanides-Nijhof (1977a)
*Aureobasidium sanguinariae*	*Gloeosporium sanguinariae*	(Ellis and Everh.) Hermanides-Nijhof	1977	*Sanguinaria canadensis leaf*	Nuttalburg, W-Virginia, USA	309386	de Hoog and Hermanides-Nijhof (1977a)
*Aureobasidium thujae-plicatae*		M. Morelet	1978	Plant	N/A	309387	Richardson and Pitkäranta (2011)
*Aureobasidium umbellulariae*	*Kabatiella phoradendri*	(Harv.) Hermanides-Nijhof	1977	*Umbellularia californica* leaves	Alpine Dam, Marine County, California, USA	309388	de Hoog and Hermanides-Nijhof (1977a)
*Aureobasidium vaccinii*		Richiteanu and Teodoru	1989	Plant	N/A	126507	Richardson and Pitkäranta (2011)

N/*A*, not available

Colonies of *Aureobasidium* that grow on malt extract agar (MEA) are initially yellow, creamy, light pink, or light brown. After a day to a few weeks, colonies become dark brown/black due to melanin-like pigments (Li et al. 2015; van Nieuwenhuijzen 2014). The hyphae and chlamydospores are the main cause of the dark pigmentation (Zalar et al. 2008). ‘Color variants’ of *Aureobasidium,* which usually have been isolated from tropical regions, produce red, yellow, orange, or purple pigments (Leathers 1986; Wickerham and Kurtzman 1975).

*Aureobasidium* species can tolerate extreme environmental conditions (Gostinčar et al. 2019) (Table 3). For instance, *A. subglaciale* shows superior resistance to UV light and heavy metals compared to other yeasts and bacteria (Liu et al. 2017). *A. pullulans* and *Aureobasidium mangrovei* are salt-resistant, the latter being able to resist salt levels up to 17% (Gunde-Cimerman et al. 2000; Zalar et al. 2008), while 20% of the cells of *A. melanogenum* survives 200 mM H_2_O_2_ (Jiang et al. 2016).

**Table 3:** Growth conditions of *Aureobasidium* species

Species name	Optimum temperature (°C)	Temperature range (°C)	Optimum pH	Survival to UV radiation (%)	Survival at 200 mM H_2_O_2_ (%)	Maximum tolerated concentration:								References
						Salt (%)	Ni^2+^ (mg/L)	Cd^2+^ (mg/L)	Cr^2+^ (mg/L)	Cu^2+^ (mg/L)	CO^2+^ (mg/L)	Pb^2+^ (mg/L)	Hg^2+^ (mg/L)	
*A. iranianum*	25	15–34	nd	nd		10	nd	nd	nd	nd	nd	nd	nd	Nasr et al. (2018)
*A. mangrovei*	25	15–37	nd	nd	Melanized cells: 20.2, non-melanized cells: 6.0	15	nd	nd	nd	nd	nd	nd	nd	Nasr et al. (2018)
*A. melanogenum*	30	10–40	nd	61.2 ± 5.1 at UV-A, 30 W for 5 min		10	nd	nd	nd	nd	nd	nd	nd	Jiang et al. (2016), Jiang et al. (2019), and Zalar et al. (2008)
*A. namibiae*	25	10–30	nd	nd		10	nd	nd	nd	nd	nd	nd	nd	Zalar et al. (2008)
*A. pullulans*	25	4–30	nd	nd		17	nd	nd	nd	nd	nd	nd	nd	Gunde-Cimerman et al. (2000), Torzilli (1997), and Zalar et al. (2008)
*A. subglaciale*	25	4–25	nd	0.8–1 at UV-B, 250 J/m2 for 15 min		10	250	50–100	400–450	400–450	300	1200	650–700	Liu et al. (2017) and Zalar et al. (2008)
*A. thailandense*	25–28	nd	nd	nd		10	nd	nd	nd	nd	nd	nd	nd	Peterson et al. (2013)
*A. zeae* (*K. zeae*)	25	15–30	7	nd		nd	nd	nd	nd	nd	nd	nd	nd	Sun et al. (2015)

*Nd*, not determined

*Aureobasidium* comprises dimorphic species that grow vegetatively by forming yeast cells and hyphae. The mode of growth depends on the species and environmental conditions (van Nieuwenhuijzen 2014). Hyphal growth of *A. pullulans* is more abundant at low cell density, while yeast cells are more abundant at high cell density (Finlay 1987; Park 1984). Hyphae of *A. pullulans* have an average width of 2–16 µm, are hyaline, smooth- and thin-walled, and become dark-brown and thick-walled when grown on MEA for a longer period (de Hoog et al. 2000; Samson et al. 2019). Yeast cells result from the division of conidia (see below), and therefore both names are used for these types of cells.

**Fig. 1:**
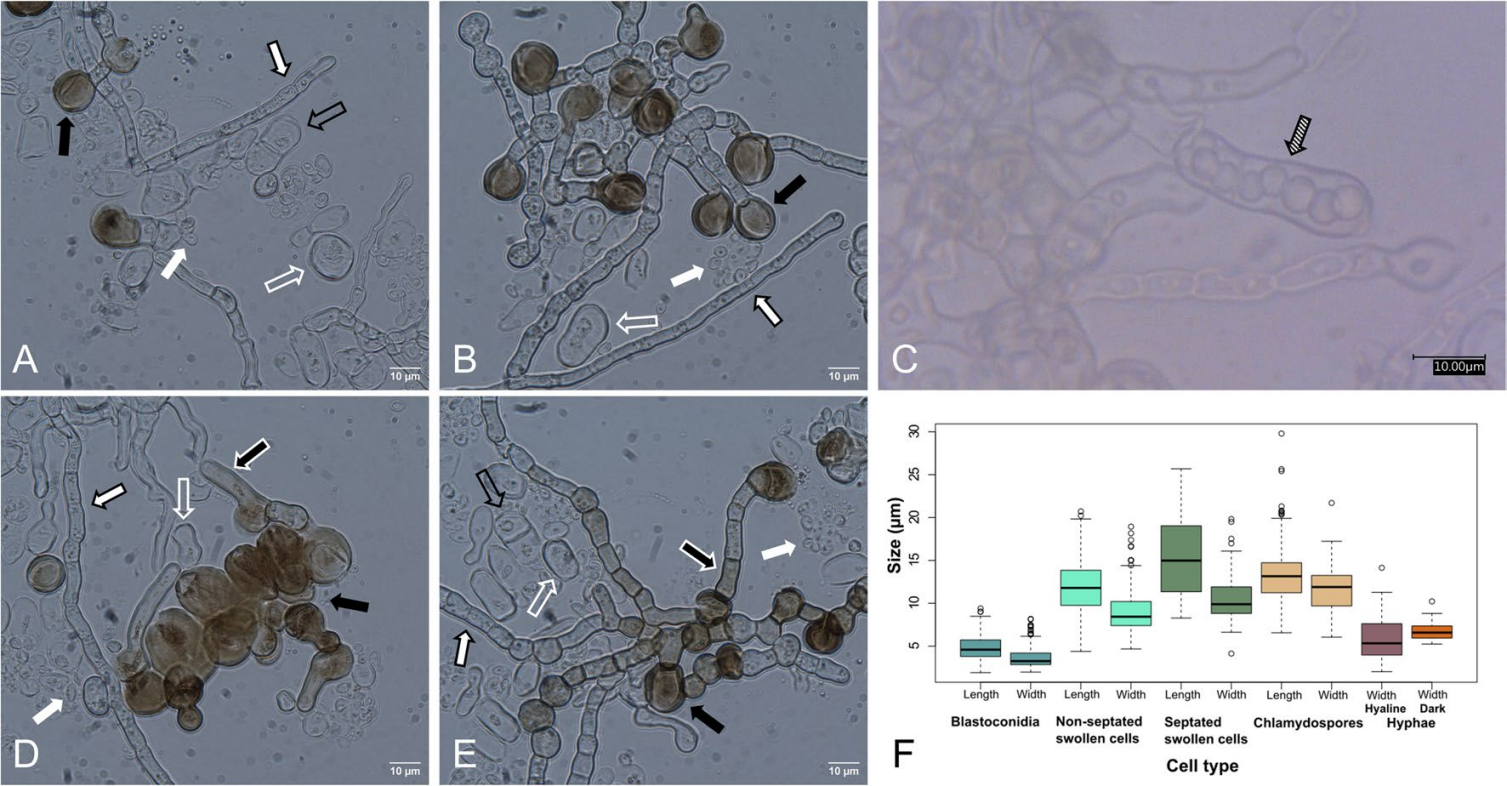
Schematic overview of cells in *A. pullulans* strain CBS 584.75 (**A**–**E**) and their dimensions (**F**). Blastoconidia or yeast cells (white arrow), non-septated swollen cells (white open arrow), septated swollen cells (black open arrow), chlamydospores (black arrow), hyaline hyphae (black lined, white filled arrow), dark hyphae (white lined, black filled arrow), and endoconidia (black striped arrow) (**A**–**E**) are the cell types distinguished in this fungal species. Arthroconidia (that could not be distinguished in liquid cultures) are also formed in this fungal species. **C** Adapted with contrast + 90 and brightness − 30

**Fig. 2:**
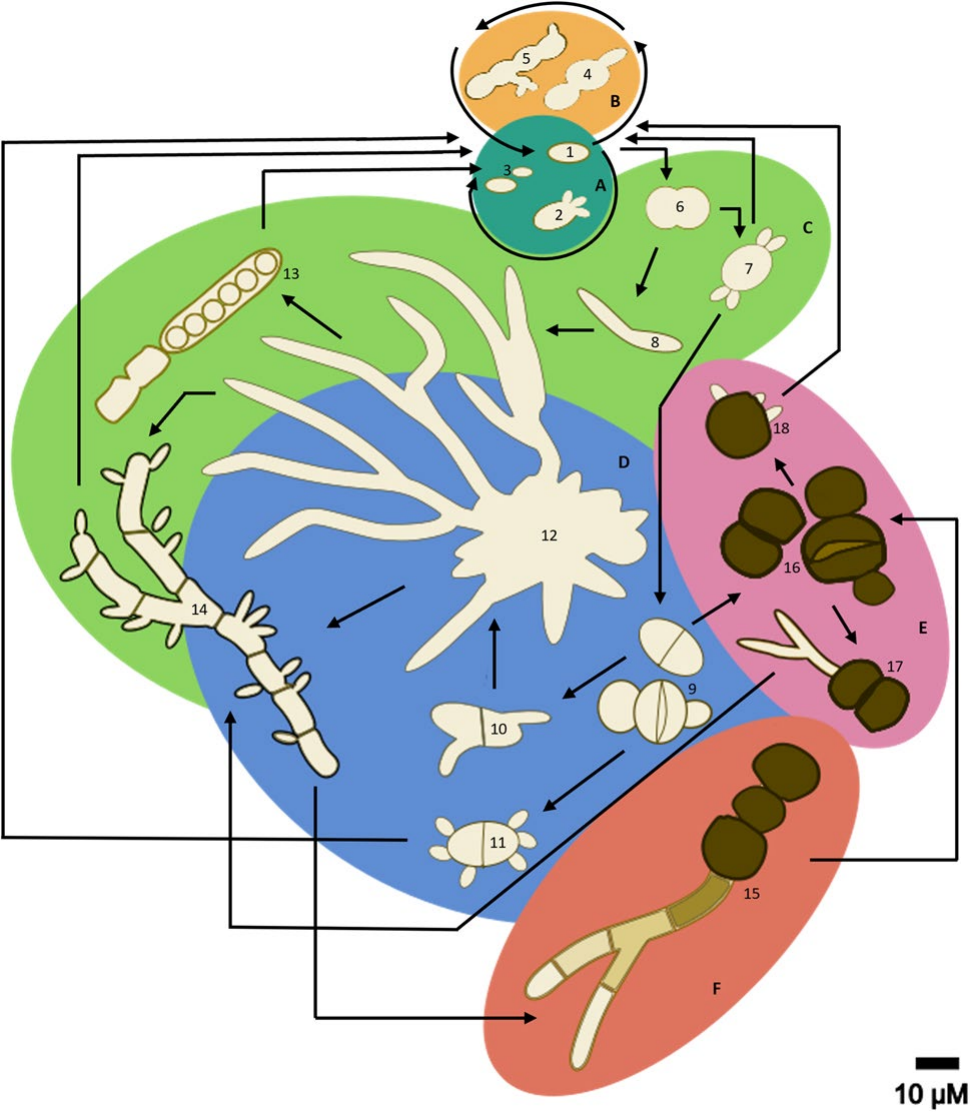
*Aureobasidium pullulans* life cycle with blastoconidia (1, 3) and blastoconidia producing other blastoconidia (2) in section A; pseudomycelium (4, 5) in section B; non-septated swollen cells (6), non-septated swollen cells giving rise to blastoconidia (7) and hyphae (8), mycelium (12), endoconidia (13), and septate mycelium producing other blastoconidia (14) in section C; septated swollen cells (9), septated swollen cells producing germ-tubes (10) or blastoconidia (11), mycelium (12), and septate mycelium producing blastoconidia (14) in section D; chlamydospores (16), chlamydospores producing germ-tubes (17), or blastoconidia (18) in section E; and chlamydospores growing on dark mycelium (15) in section F*.* Adapted from Ramos and García Acha (1975)

Although Dothideales are known to reproduce sexually, no sexual reproduction has been reported for *A. pullulans* (Humphries et al. 2017) and other *Aureobasidium* species*.* The high abundance of diploid strains in *A. melanogenum* is not considered indicative of sexual reproduction in nature. Rather, diploidy is the result of intraspecific hybridization events of haploids not being followed by meiosis or haploidization (Gostinčar et al. 2022). *Aureobasidium* does form asexual spores known as endoconidia, blastoconidia, arthroconidia, swollen cells, and chlamydospores (Figs. 1 and 2). The former four cell types are often collectively described as conidia. The formation of asexual spores depends on the species and environmental conditions (van Nieuwenhuijzen 2014). For instance, the formation of chlamydospores in *A. pullulans* is observed in a glucose medium (3% (w/v)) with a limiting nitrogen source at a low pH (Bermejo et al. 1981a; Liu et al. 2021). pH is, in fact, the key factor regulating cell morphogenesis of *A. pullulans* (Bermejo et al. 1981b; Li et al. 2009). At pH < 3 chlamydospores are produced by swollen cells (Li et al. 2009). On the other hand, blastoconidia transform into swollen cells at pH ~ 4.5, while they are stable at pH 6. These spores bud from short lateral branches of hyphae and conidiophore-like structures (van Nieuwenhuijzen 2014; Zalar et al. 2008). They are ellipsoidal, spindle-like, cylindrical, or lemon-like in shape with a size of 9–11 × 3–6.5 µm (de Hoog and Hermanides-Nijhof 1977a; Kocková-Kratochvílová et al. 1980). Swollen cells have been described to develop from blastoconidia by enlarging and by producing a thick cell wall (Campbell et al. 2004; Li et al. 2009; Pechak and Crang 1977). This cell type is globular and ellipsoid in shape and can be either septated or not, with an average size of 15 × 11 µm and 12 × 9 µm, respectively. Swollen cells not only produce blastoconidia, other (septated) swollen cells, and chlamydospores (Pechak and Crang 1977), but also germ tubes (Campbell et al. 2004). The cylindrical-shaped arthroconidia are generally bigger than blastoconidia with a length and width of 6.5–22 and 4.5–13 µm, respectively. They form through fragmentation of hyphae (Samson et al. 2019). Endoconidia are less frequently observed. They are about 6–8 × 3 µm in size and are present in intercalary cells (Zalar et al. 2008). Chlamydospores are formed from swollen cells or by hyphae and can be found in chains, as free cells, or firmly attached to hyphae (Brown et al. 1973; Dominguez et al. 1978; Pechak and Crang 1977). They have thick and melanized cell walls with single or numerous septa (Kocková-Kratochvílová et al. 1980; van Nieuwenhuijzen 2014) with an average size of 13 × 12 µm (Fig. 1). The life cycle of *A. pullulans* proposed by Ramos and García Acha (1975) consists of six subcycles, their occurrence depending on the environmental conditions (Fig. 2). In these sub-cycles, blastoconidia produce other blastoconidia or form a pseudomycelium. This pseudomycelium is formed because the daughter cells of the blastoconidia do not separate from the mother cell and continue budding, creating an aseptate chain of this type of spores. Blastoconidia can also differentiate into (non-)septated, swollen cells. These swollen cells can bud off blastoconidia, differentiate into chlamydospores, or produce germ tubes, giving rise to a septate mycelium or endoconidia. The chlamydospores can give rise to a mycelium, which in turn can produce blastoconidia and chlamydospores.

The genomes of *A. pullulans*, *A. melanogenum*, *A. subglaciale*, and *A. namibiae* contain genes that are implicated in stress tolerance, including genes encoding aquaporins, aquaglyceroporins, and alkali-metal cation transporters, proteins for the synthesis of compatible solutes and melanin, as well as bacteriorhodopsin-like proteins (Gostinčar et al. 2014). Moreover, genes encoding stress signaling pathways are present in *Aureobasidium*, including the cell wall integrity (CWI) signaling pathway, the target of rapamycin complex 1 (TORC1) signaling pathway, the high glycerol osmotic 1 (HOG1) signaling pathway, and the heat shock factor 1 (HSF1) signaling pathway (Chi et al. 2022). However, little is known about the stress resistance of most *Aureobasidium* cell types. The chlamydospores are considered resistant to desiccation and ultraviolet irradiation (Pechak and Crang 1977). It is believed that the resistant nature of chlamydospores is due to the melanin in the cell wall as well as other molecules (known as electron-dense granular material) that are present in the outermost wall layer and cross walls (Brown et al. 1973).

## Bioproducts from *Aureobasidium*

*Aureobasidium* produces many products of interest for the industry, including pullulan, β-glucan, polymalic and malic acids, melanin, lipids, biosurfactants, and liamocins. These molecules are used in agriculture, food, cosmetics, water treatment, and as pharmaceuticals and biofuels (Kim et al. 2022; Wang et al. 2022b).

### Polysaccharides

The exopolysaccharide (EPS) pullulan is produced commercially using different species of *Aureobasidium*, in particular, *A. pullulans* (Wang et al. 2022b). Pullulan consists of maltotriose units that are attached to each other via α-(1 → 6) glycosidic bonds (Singh et al. 2021). Yields up to 125.7 g l^−1^ have been achieved using agro-industrial waste (e.g., beet molasses, coconut, potato starch, and soybean) (An et al. 2017; Göksungur et al. 2004; Sheoran et al. 2012; Singh et al. 2019; Thirumavalavan et al. 2009). The highest pullulan production (162.3 g l^−1^), however, has been achieved with glucose as a carbon source (Li et al. 2023). Pullulan is a white, water-soluble, tasteless, and odorless biological binder mainly used as a food additive, functioning as a thickener, stabilizer, filler, gelling agent, and/or adhesive (Muthusamy et al. 2022; Prajapati et al. 2013). For example, it is used as a substitute for gelatin, starch, or wheat flour. Moreover, it is used in food packaging materials to prevent the oxidation of food. The viscosity of pullulan in water is not affected over a wide range of pH (2–11) and remains stable in the presence of most metal ions (Jindal and Khattar 2018; Tsujisaka and Mitsuhashi 1993). With its unique structure and being non-toxic and non-irritant to the human body, it is a potent candidate for pharmaceutical and cosmeceutical applications (Singh et al. 2023). Pullulan can be used as a carrier for the controlled release of compounds into the environment. In particular, pullulan-based conjugates have been developed for prolonged intravitreal drug release (Lu et al. 2009; Singh et al. 2023; Zhang et al. 2011). Also, active ingredients used in cosmetics and lotions can be targeted to site-specific skin layers (Nakashio et al. 1976; Singh et al. 2008). Recently, special focus has been directed toward pullulan-based biomaterials as wound dressings and skin tissue engineering scaffolds. Pullulan composites combined with other biopolymers, such as chitin, gelatin, collagen, and chitosan, are considered ideal wound dressing materials (Elangwe et al. 2023).

Along with pullulan, *A. pullulans* is known to synthesize aubasidan-like EPS. This glucan contains a core of β-1,3-linked glucopyranosyl residues, to which side chains of α-1,4-glucosyl residues are attached through β-1,6-glucosidic bonds (Singh and Saini 2012; Yurlova and De Hoog 1997). Aubasidan-like β-glucans are also produced by two non-pullulan-producing strains of *A. pullulans* (NRRL 58539 and NRRL 58543) as well as by *Aureobasidium thailandense* (Kayanna et al. 2022; Lotrakul et al. 2013). The latter produces 37.73 g l^−1^ of this polysaccharide. Adding this polysaccharide to gummy jellies results in increased color intensity, hardness, gumminess, and chewiness, as well as a decrease in springiness and cohesiveness. These properties suggest a great potential for *A. thailandense* β-glucan in the food industry (Kayanna et al. 2022). Another β-glucan formed by *A. pullulans* consists of a main chain of (1 → 3)-β-glucan and four β-(1 → 6)-d-glucosyl side chains linked to the backbone via β-(1 → 6)-glycosidic bonds every six glucose residues (Kono et al. 2017). Yields of 2.5 g l^−1^ are obtained with *A. pullulans* IMS822 KCTC 11179BP wild type (Kang et al. 2010), while 9.2 g l^−1^ is obtained with the UV mutant strain *A. pullulans* M-2 (Moriya et al. 2013). In general, β-glucans exhibit properties such as anticancer and anti-inflammatory activity, dermal wound healing, and enhancement of intestinal immune function in mice (Hu et al. 2023; Kim et al. 2023; No et al. 2021; Tanioka et al. 2013; Yun et al. 2015). Of interest, Byun et al. (2008) developed a gamma irradiation-based treatment for *Aureobasidium* β-glucan that reduces its high viscosity and poor water solubility by reducing its molecular weight.

### (Poly)organic acids

Polymalic acid (PMA) and malic acid are produced at an industrial scale by *Aureobasidium*, in particular by *A. pullulans* (Zou et al. 2013). PMA was first isolated from *Physarum polycephalum* as a compound that functions in coordination with DNA replication (Fischer et al. 1989). The α-, β-, and γ-types are distinguished, with the β-type being primarily found in *Aureobasidium* (Nagata et al. 1993; Zou et al. 2019). β-PMA is formed by ester bonds between l-malic acid (2-hydroxybutanedioic acid) monomers (Qi et al. 2021). *Aureobasidium* sp. P6 synthesizes 100.7 g l^−1^ PMA (Ma et al. 2013), while *A. pullulans* var. *pullulans* MCW produces even 152.5 g l^−1^ of this polymer (Wang et al. 2015). Obviously, these amounts are higher than those produced by *P. polycephalum* (2.7 g l^−1^) (Lee and Holler 1999). PMA has broad potential in applications such as drug delivery, biomaterials, and biodegradable plastics because of its water solubility, biodegradability, and biocompatibility (Qi et al. 2021). It can also be used in materials with controllable shape memory based on cross-linked PMA with reconfigurable permanent shapes due to further crosslinking during heat treatment (Qiu et al. 2019). Malic acid can be produced from polymalic acid by acid hydrolysis (Zou et al. 2013). It is frequently used in the food industry as an acidulant and flavor enhancer (Reddy et al. 2016). Additionally, it is applied in metal cleaning, textile finishing, and pharmaceuticals (Chi et al. 2016).

### Melanin

Many fungi synthesize melanin. These pigments are classified as 1,8-dihydroxy naphthalene (DHN) melanin, eumelanin, pyomelanin, pheomelanin, and 4-glutaminyl hydroxybenzene (GHB) melanin (Liu et al. 2022). *A. pullulans* and *A. melanogenum* mainly produce DHN melanin (Jiang et al. 2016). A total of 3.71 g l^−1^ (0.19 g l^−1^ intracellular and 3.52 g l^−1^ extracellular) of this pigment is produced by *A. pullulans* NBRC 100716 (Mujdeci 2021). It is widely applied in areas such as optical biomimetics, UV-protective lenses, food colorants, material coatings, and biomedical applications due to its functional properties related to photosensitivity, acting as a UV-light barrier, its free radical scavenging ability, antioxidant activity, and ability as a reducing and capping agent for metal nanoparticles (Campana et al. 2022; Roy and Rhim 2022).

### Fatty acids and surfactants

Fatty acids are highly produced by *A. melanogenum* P10 with a yield of 66.3 g of oil per 100 g of cell dry weight. Their composition consists of 26.7% C16:0, 1.7% C16:1, 6.1% C18:0, 44.5% C18:1, and 21.0% C18:2 (Wang et al. 2014). The transformation of corncob-derived xylose into intracellular lipid by the engineered P10 strain shows even better properties than the standard US and EU biodiesels (ASTM D6751 and EN 14214) caused by the higher cetane and lower iodine numbers (Song et al. 2022).

Surfactants have large industrial applications for their ability to lower the surface tension of water. The fact that they are produced from petroleum has triggered interest in biosurfactants (Holmberg 2001). *A. thailandese* LB01 produces a biosurfactant with a yield of 139 mg l^−1^. The biosurfactant, which has a chemical structure similar to lauric acid ester, reduces the surface tension of water from 67 mN m^−1^ to as low as 31.2 mN m^−1^. The ability of this biosurfactant to disperse crude oil highlights its potential in bioremediation (Meneses et al. 2017). *A. pullulans* is even more promising as a cell factory for biosurfactants by producing pullusurfactans F and G as well as liamocins (Brumano et al. 2017; Garay et al. 2018; Kim et al. 2022). Liamocins, also described as extracellular heavy oils, are polyol lipids belonging to the fungal glycolipid biosurfactants (Garay et al. 2018). Diverse structures of liamocins can be produced by *A. pullulans* depending on the strain and culture conditions (Leathers et al. 2015; Price et al. 2017). These glycolipids are composed of a single headgroup (d-mannitol, d- and l-arabitol, d-xylitol, l-threitol, d-sorbitol, d-galactitol, and glycerol) that is linked to three, four, or six 3,5-dihydroxy decanoic ester tail groups (Kang et al. 2022; Leathers et al. 2018). *A. pullulans* NRRL 50380 produces up to 4.4 g l^−1^ liamocins when grown on sugars and polyols (Price et al. 2017). Notably*,* a melanin-free derivative of strain NRRL 50384 (B46p14KO1) even gives a yield of 22 g l^−1^ (Leathers et al. 2018). The saturated aqueous solution of liamocins from *A. pullulans* strain CU 43 exhibits a surface tension of 27 mN m^−1^, implying these oils may have solubilizing or emulsifying properties (Manitchotpisit et al. 2011). Liamocins exhibit anticancer and antibacterial activity (Kang et al. 2022; Sałek et al. 2022) and inhibit the biofilm formation of oral streptococcal biofilms by *Streptococcus mutans* and *Streptococcus sobrinus* (Leathers et al. 2019). After hydrolysis, liamocins release 3,5-dihydroxy decanoic acids, which can be transformed into massoia lactones (Kang et al. 2022). Massoia lactones are 10, 12, and 14 carbon chain compounds (also referred to as C-10, C-12, and C-14 massoia lactones). The α,β-unsaturated δ-lactone moieties of massoia lactones are substituted at the C6 position by an alkyl chain with a variable length containing five, seven, or nine carbons (Kang et al. 2022; Rali et al. 2007). These compounds show anticancer, anti-viral, anti-inflammatory, and anti-fungal activities. Therefore, it can be used as a fungicide or pesticide in agriculture (Kang et al. 2022). For instance, massoia lactone is active against the wheat pathogen *Fusarium graminearum* (Zhang et al. 2021).

## *Aureobasidium* as plant growth promotor and biocontrol agent

Global warming results in drought and increased salinity of soils. This results in major agricultural losses (Cominelli and Tonelli 2010). Although *Aureobasidium* species are described as plant or human pathogens (Crous et al. 2011; Lee et al. 2019; Nasr et al. 2018), they are also known as plant growth promotors and biocontrol agents in various crops and fruits such as grapes, berries, apples, pears, citrus, tomato, peaches, and strawberries (Adikaram et al. 2002; Di Francesco et al. 2017; Ferreira-Pinto et al. 2006; Galli et al. 2021; Klein and Kupper 2018; Mari et al. 2012a, 2012b; Schena et al. 1999; Zajc et al. 2020). Non-volatile metabolites produced by *A. pullulans* inhibit the growth of the plant pathogen *Rhizoctonia solani* by 87.9%. Biofilm formation by *A. pullulans* strains L1 and L8 at the bean and soybean plant roots is a key factor in virulence control and plant growth stimulation (Di Francesco et al. 2021).

*Aureobasidium* species belong to the third-most common group of endophytes in desert plants. These endophytes have the capacity to increase nutrient uptake by the plant and promote resistance of the plant to pathogens and to drought, heat, and salt stress (Zhang and White 2021). The 3′-phosphoadenosine-5′-phosphatase (PAP) phosphatase ApHal2 confers resistance to sodium in *A. pullulans* (Gašparič et al. 2013). The 3′-phosphoadenosine-5′-phosphatase motif sequence META from this protein is believed to be responsible for the high tolerance to NaCl. The homolog of this protein in *Arabidopsis thaliana,* SAL1*,* lacks this motif. Therefore, a region of the ApHal2 enzyme, including the META motif, was inserted into SAL1 of *Arabidopsis thaliana* (Gašparič et al. 2013). Overexpression of this modified *SAL1* (*mSAL1*) improves the salt tolerance of the plant compared to wild-type *Arabidopsis* and plants overexpressing native *SAL1*. The msal1 plants show longer roots and larger leaf surfaces at elevated salt concentrations when compared to the sal1 and wild-type plants. Also, the wild-type plants decrease in dry weight when exposed to moderate drought stress, while the msal1 and sal1 plants even show increased dry weight in comparison with plants that are watered normally (Gašparič et al. 2013). However, severe drought results in lower dry weights in all genotypes. Also, the sal1 plants show lower revitalization ability compared with the msal1 and wild-type plants (Gašparič et al. 2013). Together, overexpression of native *SAL1* results in resistance to moderate drought stress but decreases the revitalization rate after severe drought stress. By contrast, overexpression of *mSAL1* improves salt and drought tolerance without affecting revitalization at high drought stress. These results show that *A. pullulans* or other *Aureobasidium* species can be interesting gene donors to improve the stress tolerance of plants. It is not known yet if such genes, in particular in the case of *ApHal2*, have the same effect on stress resistance when present in *A. pullulans* endophytes.

Siderophores can be used to stimulate plant growth (Di Francesco et al. 2022). These low-molecular-weight, iron-chelating compounds are produced by nearly all microbes to retrieve this metal from the environment (Chi et al. 2009; Johnson 2008). *A. pullulans* strain HN6.2 produces 1.1 g l^−1^ of siderophores (Wang et al. 2009)*.* The siderophores of *A. pullulans* strain L1 not only increase the bioavailability of Fe in the soil but also that of Mn, Cu, and Zn by 50, 31.8, 38.4, and 27.1%, respectively, after 30 days of incubation with the fungus (Di Francesco et al. 2022). This is accompanied by an increased tomato root and stem diameter of 19.1 and 27.3%, respectively.

*A. pullulans* BSS6 improves heavy metal stress resistance and remediating mechanisms in cucumber (*Cucumis sativus*) (Ali et al. 2019). Cucumber plants inoculated with *A. pullulans* BSS6 and exposed to lead and cadmium show improved plant growth and a higher content of photosynthetic pigments (chlorophyll *a* and *b* and carotenoids) compared to non-inoculated plants. *A. pullulans* BSS6 causes enhanced antioxidant activities (catalase, peroxidase, and reduced glutathione) and inhibition of lipid peroxidation during stress conditions in plants. The inoculation of *A. pullulans* BSS6 also reduces metal accumulation and alleviates metal-induced stress in plants. Finally, when added to soil *A. pullulans* BSS6 reduces the availability of lead and cadmium. These findings indicate that treatment with *A. pullulans* BSS6 is a promising phytoremediation agent for crops growing in soils polluted with these metals (Ali et al. 2019).

Biofilms are differentiated accumulations of microorganisms that are formed on surfaces surrounded by an extracellular matrix consisting of EPS (Blankenship and Mitchell 2006). The application of *A. pullulans* biofilms on winter wheat spikes inhibits the growth of pseudomonads, *Azotobacter* bacteria, and filamentous fungal pathogens (Wachowska et al. 2016). Also, biofilm production by *A. pullulans* stimulates the biocontrol activity against *Geotrichum citri-aurantii*, the causal agent of sour rot in citrus fruits, as well as other, possibly plant pathogenic, microorganisms due to niche exclusion. Biofilm production can be stimulated by the addition of 1% ammonium sulfate, which increases the antagonistic activity against sour rot and allows for better survival of *A. pullulans* in wounded sites of citrus fruits (Klein and Kupper 2018). Notably, *A. pullulans* has been found to be one of the most active endophytes cultured from fruits. It produces the highest amount (9109.19 ± 146.02 μg/g) of indole-3-acetic acid (IAA), which induces plant growth (Kachalkin et al. 2022) and, thereby, could be a great characteristic in plant growth-promoting biofilms.

## *Aureobasidium* as protective coatings in construction

In construction, the discoloration of wood due to mold growth is usually considered negative (Gobakken et al. 2010; Lie et al. 2019; Williams and Feist 1999). However, naturally grown biofilms of *A. pullulans* formed in combination with water-repellent linseed oil are, in fact, an attractive living protective wood layer (Sailer et al. 2010). Compared to traditional wood coatings, the *A. pullulans* linseed oil-based coating (from now on called Biofinish) has clear advantages in terms of sustainability and potential self-repairing abilities (Filippovych et al. 2015; 2016; Rensink et al. 2020; Sailer et al. 2010). In outdoor applications, natural Biofinish can be formed on vegetable oil-impregnated wood. This natural Biofinish consists of 26 to 34 fungal genera yet always contains species of *Aureobasidium* (van Nieuwenhuijzen et al. 2017). In terms of wood protection, liquid water uptake is prevented by the linseed oil, and together with the fungus, the wood is protected against wood-degrading microorganisms and UV light (Hernandez and Evans 2015). The latter can be explained by the high abundance of chlamydospores on the wood surface (Poohphajai et al. 2021). The industrial finish-coated wood showed superior aesthetic performance during a 3-month weathering study when compared to non-coated wood. Its surface roughness decreased, while it increased in non-coated wood. This can be explained by the local regrowth of the fungus to cover damaged spots and by the migration of the linseed oil to the wood surface, where it polymerizes (Poohphajai et al. 2021). Linseed oil consists of unsaturated linolenic (53.21%), oleic (18.51%), and linoleic (17.25%) acids, while the dominant saturated acids are palmitic (6.58%) and stearic (4.43%) acids (Gruia et al. 2012). The drying and hardening (i.e., polymerization) of linseed oil occur when the oil is exposed to air. It is a consequence of the high content of glycerol esters in linolenic acid that undergoes oxidation reactions (Juita et al. 2012). *A. melanogenum* can use linseed oil as a single carbon source (Peeters et al. 2018; van Nieuwenhuijzen et al. 2019), but not when it is cross-linked (Peeters et al. 2018). Specifically, the degree of cross-linking of the oil determines the growth of *A. melanogenum.* Assuming the same applies to *A. pullulans*, the growth of the fungus within Biofinish reduces, and eventually halts, upon cross-linking of the oil.

Adhesion is crucial for the colonization of *Aureobasidium* on any surface, including plant surfaces like wood, leaves, roots, and fruit (Blankenship and Mitchell 2006). EPS not only fill the space between cells in biofilms (Flemming et al. 2017), but these are also believed to attach cells to surfaces (Czaczyk and Myszka 2007). Adhesion of *A. pullulans* is controlled by the EPS uronic acid-based polymers and possibly pullulan as well (Bardage and Bjurman 1998; Pouliot et al. 2005). Cells harvested in the early-exponential growth phase show a lower density of uronic acid polymers but a higher adhesion to the AFM tip and a higher retention to quartz media when compared to late-exponential cells (Pouliot et al. 2005). Pullulan contributes to the adhesion of *A. pullulans* (De Bary) Arnaud blastospores on painted wood surfaces (Bardage and Bjurman 1998). In this case, early-exponential growth phase cells adhere better than late-exponential growth phase cultures of *A. pullulans* strain NRRL Y-2331–1, despite the fact that levels of pullulan are lower in the former cells. Notably, a pullulanase treatment has a minimal effect on the adhesion force, suggesting that pullulan is not involved in the adhesion of cells to silicon nitride and quartz (Pouliot et al. 2005). Unlike *A. pullulans*, *A. thailandense* is not producing pullulan. This species that is isolated from leaves and wooden surfaces (Peterson et al. 2013) may therefore produce other substances that are responsible for the adhesion to surfaces. For instance, hydrophobins could play such a role. These proteins function in hyphal attachment to hydrophobic surfaces such as those of plants. These small, cysteine-rich proteins are secreted by mycelial fungi and self-assemble at hydrophilic–hydrophobic interfaces (Wösten and Wessels 1997). For example, the hydrophobin SC3 of *Schizophyllum commune* mediates fungal attachment to hydrophobic surfaces such as Teflon. It does so by assembling into a highly surface-active protein film at the interface between the hydrophilic cell wall and the hydrophobic surface. A strain in which the *sc3* gene is inactivated shows reduced but not abolished hyphal attachment to Teflon (Wösten et al. 1994). In the absence of *SC3*, the hydrophobin-like protein SC15 mediates the attachment of *S. commune* to hydrophobic surfaces (Lugones et al. 2004). The hydrophobin genes *Aur1* and *Aur2* have been identified in *A. pullulans* strain MUCL38722, while *hfbA* and *hfbB* have been identified in *A. pullulans* (De Bary) Arnaud P268. The amino acid sequences of *aur1* and *hfbA* and *aur2* and *hfbB* are about 90% identical, and their encoded hydrophobins are predicted to belong to the class II hydrophobins, with *hfbB* being closely related to the hydrophobins of *Trichoderma* (Stenbæk 2015). The class II hydrophobins HFBI and HFBII of *Trichoderma reesei* have different properties when compared to the class I hydrophobin SC3 of *S. commune* (Askolin et al. 2006). For example, (1) in contrast to SC3, self-assembly of HFBI and HFBII at the water–air interface is not accompanied by a change in secondary structure or in ultrastructure; (2) the maximal lowering of the water surface tension occurs much faster in the case of HFBI and HFBII (instantly to several minutes) compared to SC3 (several hours); and (3) the HFBI coating has lower resistance to a hot detergent treatment than the SC3 coating. It was also shown that oil emulsions prepared with HFBI and SC3 are more stable than those prepared with HFBII and that HFBI and SC3 interact more strongly with Teflon when compared to HFBII. Surface adhesion in *Aureobasidium* may also be mediated by other proteins. As mentioned above, SC15 can partially replace SC3 in *S. commune* (Lugones et al. 2004), while hydrophobin function has been (partially) replaced by repellents in *Ustilago maydis* (Teertstra et al. 2006). A variety of proteins are surface-active, explaining why non-related proteins can mediate attachment in fungi.

## Perspective

*Aureobasidium* produces many products of interest for the industry, including enzymes, polysaccharides, and biosurfactants. These molecules have a wide range of applications. So far, the use of genetic modification has hardly been used to improve production yields. This is explained by the fact that, until recently, the efficiency of genetic modification of *Aureobasidium* was low. However, Zhang et al. (2019) developed an efficient CRISPR/Cas9-mediated genomic mutagenesis, which will be an important tool to improve the production levels of enzymes and other molecules in the future.

Apart from the use of *Aureobasidium* as a cell factory, it can also be used in coatings to protect crops or wood. For instance, *A. pullulans* is a key ingredient in commercial products. Blossom Protect (Nufarm) is used in the biological control of fire blight (Zeng et al. 2023), and Biofinish (Xylotrade) protects wood. These sustainable products replace non-sustainable chemicals and petrol-based coatings, respectively. Blossom Protect and Biofinish can be considered living materials. Nature produces a wide variety of “living” materials such as bone, wood, and tissue, while human society produces “non-living” materials like chemicals, fuels, and pharmaceuticals. Yet, researchers now start to produce living materials as well, often called engineered living materials (ELMs) (Srubar III 2021). ELMs are defined as engineered materials composed of living cells that form or assemble the material itself or modulate the functional performance of the material (Nguyen et al. 2018). These materials also contain scaffolding polymeric matrices (Rodrigo-Navarro et al. 2021). A key difference between ELMs and other biohybrid devices is that the living cells in ELMs act as material factories, whereby the cells use resources from their environment to create biopolymeric building blocks that direct and/or maintain the formation of the ELM (Nguyen et al. 2018). ELMs provide “smart” functionalities that exceed existing capabilities of conventional materials, including the adaptation to environmental conditions, different material states, and/or self-healing abilities (Nguyen et al. 2018). ELMs have been studied in the biomedical field with functions such as biosensing, wound healing, stem-cell-based tissue engineering, and drug delivery (Rodrigo-Navarro et al. 2021). Recently, ELMs have also been proposed to be implemented in the building industry to function as self-healing concrete, self-growing bricks, actuators, and energy generators, or as protective coatings and paints (Sandak 2023). As such, *Aureobasidium* biofilms can be considered a simple ELM. Its different cell types may provide different functions. For example, chlamydospores and dark hyphae produce protective melanin, while other cells provide adherence or the self-repair response. Also, certain types of cells may attract beneficial microbial partners or repel other microbes. Still, many aspects of *A. pullulans* biology have to be studied to develop this fungus as a fully functional ELM.

## Concluding remarks

*Aureobasidium* is found in soil, water, wood, and other plant materials. Various species of this genus can play a role in the transition to a sustainable economy. Their enzymes and other molecules can, for instance, be used in agriculture, construction, food, health, cosmetics, biofuel, and bioremediation. When growing in biofilms, *A. pullulans* protects crops and wood with the potential of self-repair, thereby offering a sustainable living alternative to petrol-based coatings and toxic chemicals. It should be noted that some *Aureobasidium* species, such as *A. melanogenum* are opportunistic human pathogens (Černoša et al. 2021) and are therefore not suitable for applications such as in Biofinish. Previous publications reported pathogenic *A. pullulans* strains, but these strains were likely misclassified strains of *A. melanogenum* (Gostinčar et al. 2014). Clearly, only non-pathogenic *Aureobasidium* species such as *A. pullulans* should be used in applications. To improve the use of *Aureobasidium* in a sustainable economy, future research should address which of its cell types (i.e., hyphae, yeast cells, endoconidia, blastoconidia, arthroconidia, swollen cells, and chlamydospores) and underlying mechanisms contribute to the production of enzymes or other molecules or to the formation of biofilms and their performance as a functional coating.

## Data Availability

Data will be made available on request.
